# Eliciting parental support for the use of newborn blood spots for pediatric research

**DOI:** 10.1186/s12874-016-0120-8

**Published:** 2016-02-04

**Authors:** Edwina H. Yeung, Germaine Buck Louis, David Lawrence, Kurunthachalam Kannan, Alexander C. McLain, Michele Caggana, Charlotte Druschel, Erin Bell

**Affiliations:** Division of Intramural Population Health Research, Eunice Kennedy Shriver National Institute of Child Health and Human Development, 6100 Executive Blvd, 7B03, Rockville, MD 20852 USA; Laboratory of Immunology, Wadsworth Center, Center for Medical Science, Albany, NY 12203 USA; Laboratory of Organic Analytical Chemistry, Wadsworth Center, New York State Department of Health, Albany, NY 12203 USA; Department of Epidemiology and Biostatistics, Arnold School of Public Health, Columbia, SC USA; Laboratory of Human Genetics, Wadsworth Center, New York State Department of Health, Albany, NY 12203 USA; Bureau of Environmental and Occupational Epidemiology, Center for Environmental Health, New York State Department of Health, Albany, NY 12203 USA; Department of Environmental Health Sciences, University at Albany School of Public Health, Albany, USA; Department of Epidemiology and Biostatistics, University at Albany School of Public Health, Albany, NY 12144 USA

**Keywords:** Blood spot, Consent, Newborn Screening

## Abstract

**Background:**

Biomarkers of exposures such as infection or environmental chemicals can be measured in small volumes of blood extracted from newborn dried blood spots (DBS) underscoring their potential utility for population-based research. However, few studies have evaluated the feasibility and utility of this resource; particularly the factors associated with parental consent, and the ability to retrieve banked samples with sufficient remaining volume for epidemiologic research.

**Methods:**

At 8 months postpartum, 5,034 mothers of infants born (2008–2010) in New York (57 counties excluding New York City) were asked to consent for the use of residual DBS for the quantification of cytokines and environmental chemicals. Mothers were part of the Upstate KIDS study, a longitudinal birth cohort designed to evaluate child development through 3 years of age. Information on parental and infant characteristics was obtained from birth certificates and maternal report at 4 months postpartum. Multivariate logistic regression was used to identify factors associated with parental consent and with successful retrieval of DBS.

**Results:**

Sixty-two percent (*n* = 3125) of parents consented. Factors significantly associated with consent included non-Hispanic ethnicity (odds ratio 2.04; 95 % CI: 1.43–2.94), parity (1.29; 1.05–1.57), maternal obesity (1.42; 1.11–1.80) and reported alcohol use during pregnancy (1.51; 1.12–2.06). However, these associations corresponded to small absolute differences in proportions (4 to 8 %), suggesting that the two groups remained comparable. Infant characteristics such as preterm delivery did not significantly differ by consent status among singletons and only ventilator use (OR 2.39; 95 % CI: 1.06–5.41) remained borderline significant among twins in adjusted analyses. Among consented infants, 99 % had at least one 3.2 mm punch successfully retrieved for biomarker analyses and 84 % had a full DBS circle available.

**Conclusion:**

Parental characteristics varied slightly by consent, and the availability of samples for research purposes was high, demonstrating the feasibility of this resource for population based research.

## Background

Newborn dried blood spots (DBS) can be an invaluable source of biological specimens for population based research studies requiring large sample sizes to capture rare outcomes or for establishing a baseline measure in birth cohorts for longitudinal comparisons. Technological advances have allowed for quantification of many environmental and biological markers even when sample volume is small [1]. Advances have also made it possible for their use in genetic [2] and epigenetic analyses [3]. As such, DBS analyses can provide useful information in epidemiologic studies of perinatal exposures such as dietary nutrients and infectious agents, or to validate reported exposure information (e.g., maternal tobacco use) and for understanding (epi-) genetic mechanisms [4–7].

Residual newborn screening specimens represent a unique, unbiased collection of population based material. Universal newborn screening began in 1963 in four U.S. states in response to Dr. Robert Guthrie’s discovery of the screening test for phenylketonuria, a rare metabolic disorder that if untreated early in life leads to disability [8]. Subsequently, Ireland and New Zealand implemented screening procedures and currently 67 countries across the globe now have similar screening programs [8]. Technologic advances have expanded the number of metabolic, endocrine and other conditions screened for, but vary by number in each U.S. State and across different countries [9, 10].

Despite having Newborn Screening programs in all 50 US states [8] and elsewhere across the globe, few epidemiologic studies have partnered with these programs to augment research initiatives. Controversy surrounding the ethical use of DBS and the differing policies of each state’s program may present barriers for their use in research [11, 12]. Studies have predominantly used DBS anonymously in methodological work despite some indication that parents would support their non-anonymous use [4, 13], while others have found that parents wanted to be consented every time their child’s sample was used [14]. Most recently, the Newborn Screening Saves Lives Reauthorization Act has mandated that all federally funded research on newborn DBS will require informed consent with no exception [15]. Hence, both the feasibility of eliciting parental support and the characteristics of those who provide consent compared to those who do not, is important information that could be helpful to pediatric researchers in designing future studies. This paper describes the experience of the Upstate KIDS study in eliciting parental consent and in retrieving DBS with three objectives in mind. First, we describe the process and the resources to acquire DBS for a population-based cohort to help inform future study design. Second, we sought to evaluate the sociodemographic and perinatal factors associated with parental consent for use of these specimens to identify subgroups that may be disproportionately represented. Third, we aimed to determine whether sufficient availability of residual specimen differed by participant characteristics.

## Methods

The Upstate New York Infant Development Screening Program (Upstate KIDS) [16] recruited 5,034 households to study children’s growth and development from birth through 3 years of age by mode of conception, as studies had suggested that children conceived by infertility treatment have poorer birth outcomes. Upstate KIDS enrolled infants born in Upstate New York (57 counties excluding the 5 boroughs of New York City) from 2008 to 2010 at approximately 4 months postpartum. This population based birth cohort recruited based on infertility treatment information from birth certificates [16]. All singleton mothers who conceived with infertility treatment and those conceived without infertility treatment frequency matched on region of birth (at 1:3 ratio) were recruited. Mothers of multiple births were also invited to participate irrespective of infertility treatment status. We previously compared the characteristics of non-participants (*n* = 13,490) against participants finding that non-Hispanic white race and higher socioeconomic status (i.e., education, private insurance) to be associated with participation [16]. Questionnaires assessing infant’s development were mailed at 4, 8, 12, 18, 24, 30 and 36 months. The New York State Department of Health (NYSDOH) and the University at Albany (State University of New York) Institutional Review Boards approved the Study; and served as the Institutional Review Boards designated by the National Institutes of Health for this study under a reliance agreement. All parents provided written informed consent at enrollment.

### DBS consent process

We obtained additional parental consent when infants were approximately 8 months of age for using residual specimens from the Newborn Screening Program. Specifically, we asked permission to quantify immunologic and environmental chemical biomarkers from remaining DBS. We asked for permission to assess these biomarkers of in utero infection or placental transfer of environmental chemicals for future work in assessing their relation to adverse birth outcomes [17, 18] and early childhood development [19–21]. Previous exploratory research demonstrated the feasibility of measuring these biomarkers irrespective of varying amounts of residual blood extracted from DBSs [22–25]. More specifically, DBS adipokines as measured by Upstate KIDS was associated with birth size and gestational age in similar fashion as results from cord blood measures by other studies [26].

Informed consent documents were available in both English and Spanish and were mailed to parents along with the 8-month health assessment questionnaire. Informational brochures explaining the State’s Newborn Screening Program were also included. To maximize response, the consent explicitly stated that the measurements were for cytokines and environmental chemicals, but not medications or recreational drugs, and that no genetic analyses will be performed. Voluntary participation was emphasized and made clear that the DBS consent was not a required component of study participation. The consent included telephone numbers for the site’s principal investigator and project coordinator, who were available to answer questions.

### Retrieval process

**Fig. 1:**
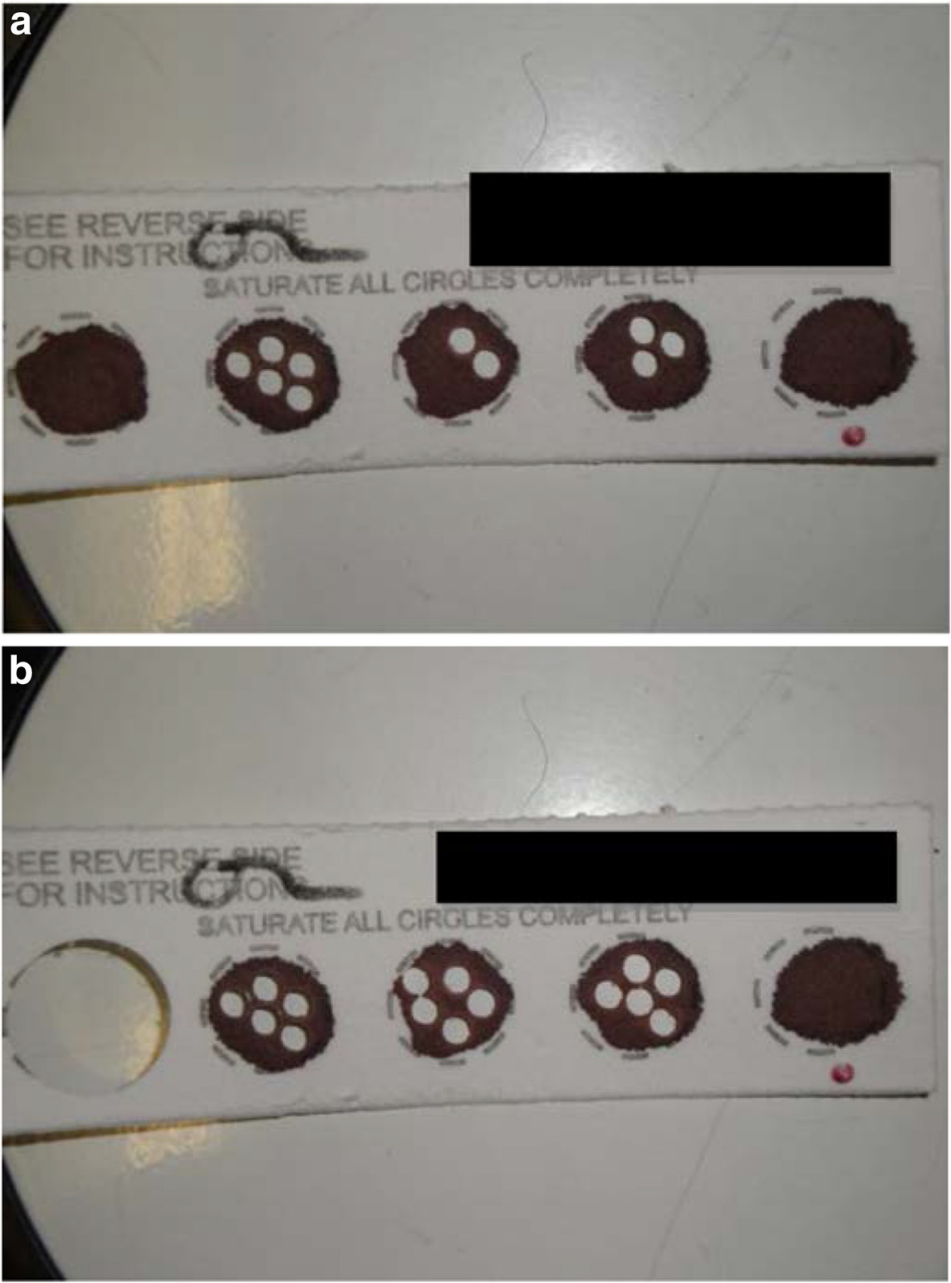
Example of before (**a**) and after (**b**) punching a DBS card. Five to six 3.2 mm punches were made by the Immunology Laboratory and 1–2 whole 16 mm punches were made by the Environmental Laboratory. One whole spot kept reserved for the Newborn Screening Program

Up to 5 blood spots could be collected from each newborn after birth by hospital staff [27]. New York State (excluding the 5 boroughs of New York City) tested for 46 disorders at the time of our sampling. Among the consented infants participating in the Upstate KIDS Study, identifying information was used to link their birth certificates to the NYS Newborn Screening Program to locate their DBS cards. Each filter paper card, containing residual blood after newborn screening, was pulled from cold storage (4 °C) by laboratory staff at the Wadsworth Center, NYSDOH.^24^ For biomarker analyses, including immunoglobulins and cytokines, five to six 3.2 mm punches were taken and assayed by the Immunology Laboratory of the Wadsworth Center. Chemical measurements required more blood volume or approximately 1–2 whole circles (~16 mm in diameter), and were retrieved by the Environmental Laboratory staff of the Wadsworth Center. By Newborn Screening policy, one whole circle had to be preserved for future medical indication. As such, specimens were only obtained if a whole circle remained. When feasible, each lab punched their samples from circles that had not been used by Newborn Screening assuming sufficient remaining sample for extended storages. Retrieval spanned from 2010 to 2013. Pulling and re-archiving the sample cards alone took almost 800 h to complete at the rate of 10 cards per hour, or the equivalent of one full time employee for six months. A few unanticipated technical issues arose, including static electricity affecting the handling of blood spots, and the time-intensive nature of extraction along with the availability of sufficient space for obtaining the punches. On average, approximately 40 cards could be punched hourly. Photos of the cards were taken before and after punching to document their location in order to assess sampling variability in future analyses (Fig. 1). We punched areas of the DBS that had no obvious signs of double spotting in that DBSs were reasonably symmetrical and the blood spot area saturated the spot based on the redness of the opposite side of the Guthrie card. Harvested DBSs had no obvious signs of smearing or a plasma ring. Each DBS circle was equivalent to approximately 50 ul of newborn blood. The 3.2 mm punches for biomarker analyses were equivalent to approximately 3.275 μl with 30 μl buffer per punch. The amount of eluted sample remaining was usually minimal to none for most analytes.

### Covariate information

Baseline characteristics were obtained either from birth certificates (i.e., maternal age, paternal age, region, plurality, parity, insurance status, and infant birth outcomes) or the mother’s baseline enrollment questionnaire obtained at 4 months postpartum (e.g., marital/partnership status). When information on a characteristic was available from both sources (i.e., maternal pre-pregnancy weight, height, race, education, infertility treatment, pregnancy smoking and alcohol use), maternal report was used as the primary source of information and birth certificates were used only if maternal report was unavailable (~2 %).

### Statistical analysis

We first focused our analyses on factors associated with household consent to use DBS. We implemented univariate *t*-test and chi-square tests, as appropriate, to identify factors associated with household consent. Factors evaluated for possible impact on consent were divided into two main areas: 1) household characteristics such as maternal/paternal age, socioeconomics, lifestyle and 2) infant factors such as birth outcomes and failure of the first developmental screen. Factors were selected based on issues that impact research consent in general (e.g., race, socioeconomic status, education), and issues that impact opting to participate in pediatric research (e.g., birth outcomes). Analyses were restricted to families who returned 8-month survey materials (*n* = 3696, 73 %) as they expressed continued response to the overall study and differences in factors by consent status identified in this group would not be a function of factors related to overall response rates. Analyses of infant factors were stratified by plurality as multiples are known to have higher risk of adverse birth outcomes than singletons. Multiples included 771 twin and 35 triplet sets. Infant factors were analyzed at the household level as there were very few parents of multiples who provided permission for one infant and not others. To do so, households with multiples were regarded as having an adverse event (e.g., NICU stay) if one (and not necessarily all infants) experienced the event, with the rationale that parents may be motivated to consent all multiples even if just one experienced an adverse event. As most multiples are preterm (<37 weeks), we additionally investigated early preterm birth defined as less than 32 weeks based on the American College of Obstetricians and Gynecologists definition. All caesarean deliveries were as reported on birth certificates regardless of indication including non-emergency or scheduled. To quantify the adjusted associations between factors and consent, multivariate logistic regression was used to estimate the odds ratio (OR) and 95 % confidence intervals (CI) for consent.

Next, we assessed parental characteristics by successful retrieval of DBS among those with consent. The units of analysis were infants and adverse birth outcomes examined including NICU stay, Apgar below 7 at 5 min, Caesarean delivery, ventilator use, preterm birth (<37 weeks) and low birth weight (<2500 g). Generalized estimating equations (GEE) were used to account for the correlation between multiples. A parsimonious model including factors that were statistically significant (*p* < 0.05) from the univariate analyses was built to identify factors associated with retrieval of a whole DBS for environmental chemical analysis. As missing covariate data was minimal (<5 %), imputation was not conducted (Table 1 footnotes). Statistical analyses were performed using SAS 9.4 (SAS Institute, Cary, NC).

**Table 1:** Baseline characteristics by Newborn Dried Blood Spot Consent Status for Upstate KIDS, New York (2008–2010)

	Among those returning infant 8-month materials (*n* = 3696)					
	No Consent (*n* = 589)		Consent^a^ (*n* = 3107)			
N (column %)	No.	%	No.	%	OR (95 % CI)	aOR (95 % CI)
Maternal age						
<25 years	83	14	415	13	1.00 (reference)	1.00 (reference)
25 - < 30 years	170	29	779	25	0.92 (0.69–1.22)	0.78 (0.56–1.11)
30 - < 35 years	179	30	1031	33	1.15 (0.87–1.53)	0.91 (0.63–1.30)
35 years or older	157	27	882	28	1.12 (0.84–1.50)	0.88 (0.60–1.31)
Singleton pregnancy	469	80	2420	78	0.90 (0.73–1.12)	0.90 (0.71–1.13)
Maternal race/ethnicity						
Non-hispanic White	460	78	2602	84	1.00 (reference)	1.00 (reference)
Non-hispanic Black	23	4	101	3	0.78 (0.49–1.23)	0.68 (0.41–1.14)
Non-hispanic Asian	11	2	91	3	1.46 (0.78–2.76)	1.64 (0.86–3.13)
Hispanic	61	10	134	4	**0.39 (0.28–0.53)**	**0.40 (0.28–0.58)**
Mixed / Other	34	6	179	6	0.93 (0.64–1.36)	0.94 (0.63–1.40)
Maternal education						
Less than high school	33	6	129	4	0.78 (0.52–1.18)	1.04 (0.61–1.78)
HS or GED equivalent	59	10	330	11	1.12 (0.82–1.54)	1.21 (0.82–1.78)
Some college	165	28	888	29	1.08 (0.86–1.35)	1.08 (0.84–1.41)
College	132	22	763	25	1.16 (0.91–1.47)	1.19 (0.92–1.52)
Advanced degree	200	34	997	32	1.00 (reference)	1.00 (reference)
Private Insurance	463	79	2476	80	1.07 (0.86–1.33)	1.07 (0.81–1.42)
Married/Living as married	512	92	2736	91	0.89 (0.64–1.23)	0.81 (0.56–1.16)
Nulliparous	314	54	1420	46	**0.73 (0.61–0.87)**	**0.71 (0.58–0.87)**
Any alcohol during pregnancy	55	9	422	14	**1.52 (1.13–2.05)**	**1.53 (1.13–2.08)**
Smoked during pregnancy	51	9	345	11	1.32 (0.97–1.79)	1.15 (0.82–1.62)
Infertility treatment						
None	396	67	2096	67	1.00 (reference)	1.00 (reference)
Drugs only	106	18	517	17	0.92 (0.73–1.17)	0.95 (0.73–1.23)
ART	87	15	493	16	1.07 (0.83–1.38)	1.15 (0.86–1.55)
Prepregnancy BMI categories						
Normal weight (BMI <25)	341	55	1598	49	1.00 (reference)	1.00 (reference)
Overweight (BMI 25–30)	132	24	757	26	1.22 (0.98–1.52)	1.25 (0.99–1.57)
Obese (BMI ≥30)	114	21	745	25	**1.39 (1.11–1.75)**	**1.43 (1.12–1.83)**

^a^Includes: 16 who could not link to newborn screening: 1 singleton, 11 twins, 4 triplets (due to unclear which child of the set); Excludes 18 who had only provided blood spot consent

Bolded for significance (*p* < 0.05)

aORs adjusted for all other household/maternal factors in the Table

Total N for the models were reduced due to missing information: 1 for specific type of infertility treatment, 2 for insurance status, 126 for marital status, 28 for parity, 2 for alcohol, 2 for smoking, and 9 for BMI

## Results

**Fig. 2:**
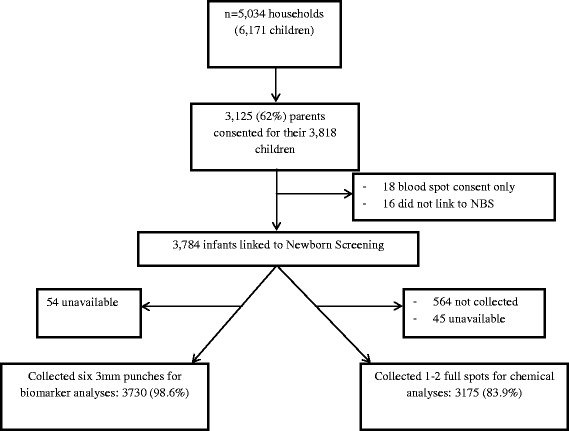
Flowchart outlining newborn dried blood spot retrieval of punches for Upstate KIDS

Sixty-two percent (*n* = 3,125) of all enrolled households (*n* = 5,034) consented to DBS use (Fig. 2). This proportion did not differ by plurality with 60.9 % households of multiples (*n* = 687 of 1129) providing consent. Parents of multiples predominantly provided consent for all or none of their infants with few discordant (i.e., 17 twins and 3 triplets had discordant consent status from their siblings). All but 16 infants whose parents consented were successfully linked to the Newborn Screening Program database. The inability to link was due primarily to difficulties in distinguishing multiple birth sibships in a set from one another (i.e., 15 of 16 were twins/triplets). DBS were collected at a median of 2 days postpartum (inter quartile range of 2–3 days). Of infants who linked, 99 and 84 % had sufficient remaining specimen for the study by the Immunology and Environmental laboratories, respectively; that is, 99 % had at least 3 mm diameter of space to punch and 84 % had a full circle available.

Of the 3125 households with a returned consent, 3107 (99.4 %) returned other 8-month materials. As this high proportion indicated that consent is conditional on continuing response to the overall study, Tables 1 and 2 show the differences in characteristics by consent status restricted to those who returned their infant 8-month questionnaire. Among those who provided questionnaire responses, 84 % agreed to DBS analyses. Few household/maternal characteristics were identified as being associated with consent after mutual adjustment for other covariates (Table 1). Women who were nulliparous or Hispanic were less likely to consent whereas those who consumed any alcohol in pregnancy or were obese were more likely to significantly consent compared to their counterparts. Despite the significant odds ratios for these characteristics, absolute differences ranged from 4 to 8 %, suggesting that the two groups remained comparable.

**Table 2:** Birth outcomes of consented participants for Newborn Dried Blood Spot analyses stratified by plurality among households returning the 8-month packets for Upstate KIDS, New York (2008–2010)

	Singletons (2889 households)			Multiples (807 households)		
	Non-consent	Consent	*p*-value	Non-consent	Any Consent	*p*-value
N (row %)	469 (16)	2420 (84)	n/a	120 (15)	687 (85)	
Ventilator	9 (2)	84 (3)	0.08	8 (7)	124 (16)	0.002
NICU	29 (6)	174 (7)	0.43	40 (33)	300 (44)	0.03
Cesarean section (any)	187 (40)	931 (38)	0.57	103 (86)	536 (78)	0.05
Preterm (<37 weeks)	31 (7)	185 (8)	0.44	57 (48)	374 (55)	0.22
Early preterm (<32 weeks)	6 (1)	43 (2)	0.45	10 (4)	129 (11)	0.006
Low birth weight (<2500 g)	21 (4)	124 (5)	0.56	74 (62)	423 (62)	0.93
Deaths	n/a	n/a		1 (1)	31 (4)	0.07

N (column %) unless indicated. *N* = number of households/mothers with conditions specified as “any” or “all” multiples as there were few discordant between twins in consent (i.e., 17 twins had discordant consent status with their co-twin). There were few triplets (33 sets consented, 2 not) and quadruplets (none consented, 1 set consented) so data not shown

p-value by chi-square or Fisher’s exact test (for *n* < 5)

As for infant outcomes, analyses stratified by plurality showed that a greater percentage of consents were received from households of multiples having adverse birth outcomes (i.e., ventilator use, NICU stay, early preterm birth) than homes of multiples without adverse outcomes. These associations were not evident among households with singleton births. After adjustment for significant parental characteristics from Table 1 (i.e., race, alcohol use, parity, BMI), only ventilator use (OR 1.95, 95 % CI: 1.13–3.36) remained a significant factor for consent among multiples whereas NICU stay (OR 1.12; 0.81–1.56) and early preterm birth (OR 1.51; 0.82–2.78) did not remain significant. There were no significant associations for singletons in adjusted models (*p* > 0.10) (data not shown).

Among infants with consent received for DBS analysis (*n* = 3782), very few factors were significantly associated with their spots not being retrieved (*n* = 609) by the Environmental Lab which required at least one whole DBS circle (Table 3). In multivariate analysis, having an infant who required a NICU stay compared to not and less than high school education compared with advanced degree remained significant factors for inability to retrieve a whole circle.

**Table 3:** Odds ratios (95 % CI) to identify factors for newborn dried blood spots not being collected among infants with parental consent in Upstate KIDS, New York (2008–2010)

N (collected/not collected)	3782 (3173/609)^a^	
	Crude OR for not collected	aOR (95 % CI)
Multiple birth	1.21 (1.01, 1.44)	1.05 (0.85, 1.31)
Private insurance	0.82 (0.68, 0.98)	0.97 (0.78, 1.20)
Maternal race/ethnicity		
Non-hispanic white	1.00 (reference)	1.00 (reference)
Non-hispanic Black	0.68 (0.39, 1.20)	0.61 (0.35, 1.06)
Non-hispanic Asian	0.99 (0.61, 1.60)	1.03 (0.64, 1.67)
Hispanic	1.39 (1.00, 1.93)	1.20 (0.86, 1.67)
Mixed race or ethnicity / Other	0.95 (0.66, 1.36)	0.92 (0.65, 1.31)
Maternal education		
Less than high school	1.78 (1.31, 2.42)	1.68 (1.19, 2.37)
HS or GED equivalent	1.20 (0.92, 1.57)	1.19 (0.90, 1.59)
Some college	1.03 (0.84, 1.27)	1.03 (0.83, 1.27)
College	0.78 (0.62, 0.99)	0.77 (0.61, 0.98)
Advanced degree	1.00 (reference)	1.00 (reference)
NICU	1.44 (1.21, 1.71)	1.33 (1.05, 1.67)
Ventilator (any)	1.28 (1.03, 1.60)	1.18 (0.93, 1.50)
Preterm (<37 weeks)	1.27 (1.05, 1.53)	0.93 (0.70, 1.23)
Low birth weight (<2500 g)	1.27 (1.08, 1.49)	1.14 (0.92, 1.42)

^a^ 2 missing insurance status

## Discussion

Our experience in New York State (excluding New York City) demonstrates that residual DBS are a feasible way to obtain biological samples to augment a population based cohort study. Over 60 % of the cohort consented to DBS use with the proportion increasing to 84 % among those who indicated continued participation in the study through return of other 8-month materials. With the Newborn Screening Saves Lives Reauthorization Act of 2014 requiring consent for use of residual DBS, our results are reassuring that the majority of parents would be supportive of such research efforts and that obtaining consent should not be a deterrent for leveraging this valuable resource. We also demonstrated highly successful retrieval with success for 99 % of consented infants when only 3 mm punches are needed and 84 % even when a whole blood spot circle is required for analyses.

Policies on using DBS vary widely and multiple international organizations wrestle with their ethical use [11, 28–31]. Debate continues whether all uses (even when de-identified) should require parental consent [31]. A study using focus groups of expectant parents identified typical concerns with using residual DBS in research, including understanding the use of the DBS and the methods for safeguarding the information, especially in cases where it may cause potential discrimination for their children [13]. On the other hand, parents from the focus group wanted to know the results and viewed anonymous research as actually less beneficial [13]. However, the acceptability of anonymous research remains high. A new consent protocol was introduced in Victoria, Australia which asked parents’ permission to use remaining newborn screening samples in de-identified health research [32]. For over 77,000 newborns, only 6.5 % of parents declined the use of their child’s residual DBS in secondary research and another 1.4 % did not respond [32]. Our finding that the majority of parents are willing to provide consent concurs with their study. However, one weakness of our study was that we did not actively reach out to parents with missing DBS consents to confirm their opinions of DBS use. Face-to-face contact may have also increased our response as other studies have found with respect to recruitment [33].

Our success was due to crucial partnerships with the New York State Department of Health including Newborn Screening and the other laboratories of the Wadsworth Center; our experience may not be generalizable to all states as state policies on their use vary widely. Given the importance of involving stakeholders in research, as exemplified by the establishment of the Patient-Centered Outcomes Research Institute (PCORI) [34], such collaborations will likely increase in the future. Bringing together parents, departments of health and research institutions could assist with establishing neonatal research priorities. A national feasibility study attempting to retrieve DBS from across the US found significant geographic variability in DBS retrieval rates [35]. Of note, New York was one of the states where investigators had success in retrieving DBS samples [35]. The study found a similar consent rate (58 %) as ours (62 %) when restricted to families that were reached. These consent rates support findings from another survey [36] and indicate that the majority of the population condones the use of residual DBS for research purposes regardless of whether the family had a sick child (e.g., pediatric cancer [35]) or a more general population (e.g., Upstate KIDS). Other states may not release DBS for research use and some states do not retain specimens for more than a year [28]. In such states, research using residual DBS would have to seek consent prior to or just after birth to ensure prolonged storage, allowing for later retrieval. In our study, retrieval occurred 1–5 years after birth. Recent recommendations to include DBS card serial numbers on birth certificates [37] may make retrieval for studies easier.

We acknowledge that many European cohort studies have successfully collected cord blood samples for their birth cohorts and may not have to rely on stored blood spots [38]. However, for areas of the world where the cost or the logistics of collecting specimens are prohibitive (e.g., for home births), we demonstrate that DBS is an alternative acceptable to most parents. Many international research studies have also turned to DBS due to its minimal invasiveness and feasibility without relying on trained phlebotomists [39]. Moreover, the infrastructure for the collection is already established, making it a great resource for population based studies. The Wadsworth Center also has established methods to measure immunological [22] and environmental chemical markers [23–25] and successfully completed the analysis in the samples collected for the Upstate KIDS study, demonstrating feasibility. Of note, cord blood collected at delivery may be different from that obtained on blood spots that are typically collected 2–3 days postpartum. However, cohort studies are also moving towards collecting blood spots longitudinally throughout childhood due to their greater acceptability over venipuncture among healthy children as well as less logistical burden than serum/plasma collection requiring centrifugation [40].

Over 80 % of parents who returned the 8-month study questionnaires consented to the use of DBS. Consent may differ if we had not explicitly excluded genetic or pharmacologic tests on the consent forms, described pilot tests for detecting new disorders as part of newborn screening, or if parents were not part of an ongoing cohort study. As participants had self-selected into the parent study, they may have had a more positive view of health research or might have also wanted additional contact with the medical system which could bias them towards consenting. We identified very few factors significantly associated with consent, including Hispanic race, parity, alcohol use, and maternal obesity. We had a predominantly white (80 %) cohort, and our ethnic difference in consent, based on <200 Hispanic households, may not be generalizable to all studies. However, among our participants, factors that are typically regarded as barriers to participation and possible reasons for ethnic disparities [41], such as access to research, language, or time were eliminated by availability of Spanish language materials and return of materials by mail rather than clinic visit. Our findings may not be broadly generalizable to all population based studies due to low initial response rate for participating in the study (22 %) [16] and the attrition (27 %) experienced at the time of DBS consent. One recent meta-analysis has contradicted the perception of minority mistrust, finding that minorities are as willing, or even more willing to participate in research studies than non-Hispanic whites, although considerable heterogeneity between studies was acknowledged [41]. Greater understanding of the rationale for not consenting may be helpful for future studies especially among minorities.

## Conclusion

Researchers should consider residual DBS as a source of biological specimens from newborns for population-based cohort studies. We presented the technical efforts used for such an undertaking so that future researchers can plan for the necessary resources required. Newborn blood spot registries offer novel opportunities for research aimed at understanding children’s health, particularly in relation to in utero exposures that can be quantified using banked specimens. Undoubtedly safeguards of this resource need continual evaluation, given concerns previously raised for their ethical use [11]. Nevertheless in our study, the majority of parents agreed with this practice. We demonstrate in New York State (excluding New York City) that retrieval of one whole circle from filter paper card was successful for over 80 % of infants whose parents consented to the study, and almost all (99 %) had a sufficient number of 3.2 mm punches available.

## Abbreviations

CI: confidence interval
DBS: dried blood spots
NICU: neonatal intensive care unit
NYSDOH: New York State Department of Health
Upstate KIDS: Upstate New York Infant Development Screening Program
OR: odds ratio
